# Integrative metabolomic and network pharmacological analysis reveals potential mechanisms of *Cardamine circaeoides* Hook.f. & Thomson in alleviating potassium oxonate-induced asymptomatic hyperuricemia in rats

**DOI:** 10.3389/fphar.2023.1281411

**Published:** 2023-11-02

**Authors:** Yingli Zhu, Songrui Di, Yipeng Li, Weican Liang, Jinlian Liu, Reyisai Nuermaimaiti, Wenting Fei, Chun Wang, Linyuan Wang, Jianjun Zhang

**Affiliations:** ^1^ School of Chinese Materia Medica, Beijing University of Chinese Medicine, Beijing, China; ^2^ School of Traditional Chinese Medicine, Beijing University of Chinese Medicine, Beijing, China

**Keywords:** *Cardamine circaeoides* Hook.f. & Thomson, *Cardamine circaeoides* Hook.f. & thomson aqueous extract, hyperuricemic, potassium oxonate, metabolomics, network pharmacology

## Abstract

*Cardamine circaeoides* Hook.f. & Thomson (CC), a herb of the genus *Cardamine* (family Brassicaceae), has a rich historical usage in China for both culinary and medicinal purposes. It is distinguished by its remarkable ability to hyperaccumulate selenium (Se). CC has demonstrated efficacy in the prevention of metabolic disorders. However, investigations into the effects of CC on asymptomatic hyperuricemia remain scarce. The objective of this study is to elucidate the mechanism by which CC aqueous extract (CCE) exerts its anti-hyperuricemic effects on asymptomatic hyperuricemic rats induced by potassium oxonate (PO) by integrating metabolomics and network pharmacological analysis. Asymptomatic hyperuricemia was induced by feeding rats with PO (1000 mg/kg) and CCE (0.75, 1.5, or 3 g/kg) once daily for 30 days. Various parameters, including body weight, uric acid (UA) levels, histopathology of renal tissue, and inflammatory factors (IL-1β, IL-6, IL-8, and TNF-α) were assessed. Subsequently, metabolomic analysis of kidney tissues was conducted to explore the effects of CCE on renal metabolites and the related pathways. Furthermore, network pharmacology was employed to explicate the mechanism of action of CCE components identified through UPLC-Q-TOF-MS analysis. Finally, metabolomic and network-pharmacology analyses were performed to predict crucial genes dysregulated in the disease model and rescued by CCE, which were then subjected to verification by RT-qPCR. The findings revealed that CCE significantly inhibited the UA levels from the 21st day to the 30th day. Moreover, CCE exhibited significant inhibition of IL-1β, IL-6, IL-8, and TNF-α levels in renal tissues. The dysregulation of 18 metabolites and the tyrosine, pyrimidine, cysteine, methionine, sphingolipid, and histidine metabolism pathways was prevented by CCE treatment. A joint analysis of targets predicted using the network pharmacology approach and the differential metabolites found in metabolics predicted 8 genes as potential targets of CCE, and 3 of them (PNP gene, JUN gene, and ADA gene) were verified at the mRNA level by RT-qPCR. We conclude that CCE has anti-hyperuricemia effects and alleviates renal inflammation in a rat model of hyperuricemia, and these efficacies are associated with the reversal of increased ADA, PNP, and JUN mRNA expression in renal tissues.

## Graphical Abstract

**Figure:**
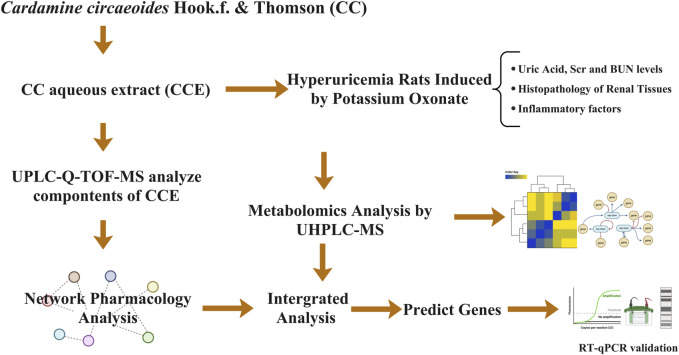
The fabrication of bone tissue engineering scaffolds including bioactive factors by low-temperature deposition manufacturing and its application for bone defect repair.

## 1 Introduction

Asymptomatic hyperuricemia is a non-pathological condition characterized by elevated levels of uric acid (UA) in the blood, without the presence of gout or nephritis (Popa-Nita et al., 2008). Its global prevalence rate is between 10% and 20% (Yip et al., 2020). Once the concentration of UA, which is the end product of purine metabolism, exceeds normal levels (Saito et al., 2021), it results in hyperuricemia (Dewulf et al., 2022). While UA possesses antioxidant properties at normal physiological levels (Ames et al., 1981), higher concentrations, especially soluble UA, exhibit pro-inflammatory effects (Al Shanableh et al., 2022). Joosten et al. showed that soluble UA activates inflammatory responses by promoting the generation of intracellular reactive oxygen species (Joosten et al., 2020). Additionally, Braga et al. (Braga et al., 2017) demonstrated that soluble UA activates the NLRP3 inflammasome, leading to the release of pro-inflammatory interleukin-1β (IL-1β). Furthermore, UA inhibits NO production (Hisatome et al., 2021) and induces the secretion of interleukin-6 (IL-6) (Guo et al., 2019), interleukin-8 (IL-8) (Liang et al., 2015), and tumor necrosis factor-alpha (TNF-a) (Lobo et al., 2013), which contributes to an inflammatory response.

Furthermore, the persistent elevation of UA concentrations increases the risk of chronic inflammatory diseases such as gout and diabetes (Borghi et al., 2020). The current clinical treatment of hyperuricemia primarily relies on pharmacological interventions supplemented by diet and lifestyle modifications. However, prolonged usage of medications aimed at reducing UA levels may lead to adverse effects such as severe allergic reactions and kidney damage (Singer and Wallace, 1986; Lee et al., 2021).

*Cardamine circaeoides* Hook.f. & Thomson (CC) is a herb of the genus *Cardamine* (family Brassicaceae) (Liu et al., 2022). In China, *Cardamine* has been utilized for hundreds of years as both a culinary ingredient and a medicinal herb. Its historical usage can be traced back to the Ming Dynasty, where it was documented in *Ye Cai Pu* by Wang Pan. According to this text, the whole herb is a diuretic, relieves pain, and soothes cough and breathlessness. Local doctors also used it to treat menstrual irregularities in women, to clear heat and dampness, and to alleviate diarrhea. CC is known for its ability to accumulate selenium (Se) in its leaves and roots (Zhu et al., 2019). It was first discovered in 1957 in Enshi, China (Rao et al., 2020). Selenium is an essential element (Kiełczykowska et al., 2018) and offers various beneficial effects, including antioxidant properties, and improvement of metabolic syndrome. Consequently, CC holds significant value as a traditional herbal medicine and warrants further investigation.

Currently, the majority of research on CC has primarily focused on selenium-enriched peptides, with a limited number of studies on their chemical composition and pharmacological activities; studies have shown that selenium-enriched peptides derived from CC can mitigate oxidative stress and inflammatory response, thereby preventing obesity and metabolic disorders induced by high-fat diets (Yu et al., 2020). However, the effects of CC on asymptomatic hyperuricemia induced by potassium oxonate (PO) have yet to be investigated. Therefore, we hypothesized that CC aqueous extract (CCE) may have an anti-hyperuricemic effect associated with its anti-inflammatory activity. Additionally, the specific components of CCE have not been extensively studied and identified. This study aims to identify its specific ingredients.

Metabolomics can provide a rapid approach for identifying the overall changes in metabolite associated with the anti-hyperuricemic effects of CCE (Xiang et al., 2021). Additionally, network pharmacology can provide insights into the molecular-level mechanisms of CCE by exploring the interactions between multiple compounds and targets (Yan et al., 2022). Therefore, the combination of network pharmacology and metabolomics could help to identify the key gene targets and gain a deeper understanding of the underlying mechanisms.

In this study, we investigated the pharmacological effects of CCE in lowering UA levels and studied the pathology and biochemical alterations of the UA-induced inflammatory state. Network pharmacology and metabolomics were then employed to gain comprehensive insights into the potential mechanisms of CCE’s UA-lowering effects. Finally, by combining the findings from the two analyses, we could predict and validate essential genes and elucidate the mechanisms through which these gene targets operate.

## 2 Methods

### 2.1 Preparation of CCE

CC was dried and ground to a powder (Se content was 300 ppm, donated by Enshi Autonomous Prefecture Academy of Agriculture Sciences, Enshi, Hubei Province, China). The powdered samples were then subjected to extraction with 10–20 times the volume of water for a duration of 20–30 min. The resulting solution was filtered, vacuum-concentrated, and stored at −20°C (hereafter referred to as CCE).

### 2.2 Composition analysis of CCE

To determine the composition of CCE, UPLC-Q-TOF-MS analyses were employed, following the methodology described in a previous paper (Zhu et al., 2023). The specific parameter setup in this study can be found in the Supplementary Materials TS1, TS2.

### 2.3 Animals and CCE administration

SD rats (male, SPF, 180–200 g) were provided by Vital River Laboratory Animal Technology Co., Ltd. (Beijing, China). The rats were housed in an SPF laboratory animal room with access to standardized animal food and water *ad libitum*. All the procedures were conducted in accordance with the guidelines approved by the Medical Ethics Committee of Beijing University of Chinese Medicine (No. BUCM-4-2022033103-1069). To induce the model, PO was administered at a dosage of 1000 mg/kg, and CCE was administrated daily by gavage for 30 days. The rats were randomly divided into six groups (eight rats per group): The control group (Control), PO-induced model group (Model), and allopurinol treatment group (positive group, PG) received a dose of 27 mg/kg; the low-dose CCE group (L-CCE) received a dose of 0.75 g/kg; the medium-dose CCE group (M-CCE) received a dose of 1.5 g/kg; and the high-dose CCE group (H-CCE) received a dose of 3 g/kg. The Model group was given PO. The PG and CCE groups were given PO first, followed by administration of PG and CCE after a 4-h interval.

### 2.4 Serum biochemical assay

Blood samples for measurement of UA levels were collected from the orbital venous plexus at days 0, 7, 14, 21, 28, and 30. The samples were allowed to coagulate for 1 h at room temperature, and then the serum was separated by centrifugation (3,000 rpm for 10 min at 4°C). The levels of serum UA levels (measured using kit no. 120600B produced by Prodia Diagnostics, Germany), serum creatinine (Scr) (measured using kit no. 141122015 produced by Shenzhen Mindray Bio-Medical Electronics Co., Ltd.), and blood urea nitrogen (BUN) (measured using kit no. 0407608 produced by Prodia Diagnostics, Germany) were measured using a commercially available Biochemical kit on an automated biochemical analyzer (Hitachi 7600, China) following the manufacturer’s protocol on the 30th day.

### 2.5 Renal tissues biochemical assay

Renal tissues were allowed to coagulate for 1 h at room temperature, and then the tissues were separated by centrifugation (3,000 rpm for 10 min at 4°C). The levels of inflammatory factors (IL-1β, IL-6, IL-8, and TNF-α levels) were measured using a commercially available ELISA assay kit on an automated biochemical analyzer (TECAN, Switzerland) following the manufacturer’s protocol. The specific kits used were IL-1β (E20220607-30206A), IL-6 (E20220607-30219A), IL-8 (E20220607-30221A), and TNF-α (E20220607-31063A), purchased from Shanghai Mlbio Co., Ltd.

### 2.6 Histopathology analysis

The kidney tissues of the rats were first fixed with 10% formalin and subsequently washed with phosphate buffer solution (PBS). Then, after dehydration in a graded ethanol solution, the samples were embedded in paraffin. Tissue section was performed following a standard protocol, and tissue slices were stained with hematoxylin-eosin (HE) for routine morphological assessment.

### 2.7 Metabolomics analysis

#### 2.7.1 Metabolites extraction

500 µL of extraction solution (methanol: acetonitrile: water = 2:2:1) was mixed with 25 mg kidney sample, which was then homogenized at 35 Hz for 4 min, followed by sonication in ice water (3 × 5 min) (Zhao et al., 2022). The sample was then incubated at −40°C for 1 hour before centrifugation (12000 rpm, 15 min, 4°C) (Abdelhafez et al., 2020). The supernatant was collected for further analysis. Additionally, quality control (QC) samples were prepared to ensure the accuracy and reproducibility of the analysis.

#### 2.7.2 LC-MS/MS analysis

An ultra-high performance liquid chromatography system (Vanquish, Thermo Fisher Scientific) was used to perform LC-MS/MS analyses, which included a UPLC BEH Amide column (2.1 mm × 100 mm, 1.7 μm) and a Q Exactive HFX mass spectrometer. The mobile phase contains two parts: (A) ammonium acetate (25 mmol/L) mixed with ammonium hydroxide aqueous solution (25 mmol/L, pH = 9.75), and (B) acetonitrile. The temperature of the autosampler was set at 4°C. The injection volume was 2 μL. The ESI source conditions were as follows: 1) Auxiliary gas flow rate of 25 Arb, 2) sheath gas flow rate of 30 Arb, 3) MS/MS resolution 7,500 Units, 4) collision energies of 10/30/60 (NCE mode), 5) capillary temperature of 350°C, 6) injection voltages of 3.6 kV (positive) or −3.2 kV (NCE mode), and 7) full MS resolution of 60,000.

#### 2.7.3 Data pre-processing and analysis

The acquired raw data underwent conversion to the mzXML format using ProteoWizard. Subsequently, data preprocessing steps including detection, extraction, comparison, and integration were performed using R. An in-house MS2 database (BiotreeDB) was developed and was then applied for metabolite annotation with the threshold set at 0.3. Principal component analysis (PCA) was employed. Furthermore, the Orthogonal Projection Latent Structure Discriminant Analysis (OPLS-DA) was carried out by SIMCA software (version 16.0.2).

#### 2.7.4 Metabolite identification and enrichment analysis

Differential metabolites were identified and compared between the Control, Model, and H-CCE groups with Variable Importance in Prediction (VIP) > 1 and *p* < 0.05. MetaboAnalyst 5.0 (https://www.metaboanalyst.ca/) and the KEGG database were employed to analyze metabolic pathways.

### 2.8 Network pharmacology analysis

#### 2.8.1 Target prediction

All the identified CCE compounds were retrieved through PubChem (https://pubchem.ncbi.nlm.nih.gov/) in SMILES format and were then subjected to target prediction using Swiss Target Prediction (https://www.swisstargetprediction.ch/). The labeled targets were analyzed by the UniProt database (https://www.uniprot.org/) and standardized to official gene symbols.

#### 2.8.2 Collection of hyperuricemia genes

To collect data on disease genes related to hyperuricemia, a search was conducted using the keyword “hyperuricemia” in GeneCard (https://www.genecards.org/). Predicted targets and disease targets were then screened via the Venn tool (version 2.0.2), and the intersections were considered potential targets.

#### 2.8.3 Construction of a PPI network and analysis of modules

Targets were submitted to STRING (version 11.0) to elucidate the relationships between the identified targets and construct PPI networks. For more convincing genes, a threshold of “medium confidence” (>0.400) was set. The acquired PPI relationship data were visualized using Cytoscape (version 3.9.1). Additionally, the Molecular Complex Detection (MCODE) plugin was utilized, which allows analysis of the core sub-network in the PPI network and downstream enrichment analysis.

#### 2.8.4 Functional enrichment and pathway analysis

DAVID (https://DAVID.ncifcrf.gov/, version 6.8) was used for Gene Ontology (GO) and Kyoto Encyclopedia of Genes and Genomes (KEGG) enrichment. The DAVID database in conjunction with the FunRich software (https://www.funrich.org/, version 3.1.4), was employed to analyze and visualize the gene ontology results for biological process (BP), molecular function (MF), and cellular component (CC) ontologies, respectively. Additionally, KEGG pathways were also analyzed and annotated.

### 2.9 Predicted key gene targets

MetaboAnalyst 5.0 (https://www.metaboanalyst.ca/) was used to construct a gene-metabolite network. Then, the key gene targets of CCE were predicted. Based on the genes associated with metabolites in the network, these genes were considered to be the key gene targets.

### 2.10 Key gene targets validation

Real-time fluorescence quantitative polymerase chain reaction (RT-qPCR) was used to examine the expression level of the predicted key gene targets in 3 kidney tissues from the H-CCE group. The experimental protocol for RT-qPCR followed the method described in a previous study (Fei et al., 2022). The primers used for target genes in this study can be found in Supplementary Materil TS3.

### 2.11 Statistical analysis

The effect of each group was analyzed by GraphPad Prism 9 (GraphPad Software, Inc., San Diego, United States) and SPSS 20.0 (SPSS Inc., IBM Corp., Armonk, NY, United States). All data were expressed as means ± SEM. Statistical analysis was performed using a one-way analysis of variance, followed by the Least-Significant Difference (LSD) *post hoc* test or Dunnett T3 test for comparison of multiple groups. Statistical significance was considered at *p* < 0.05.

## 3 Results

### 3.1 Compounds analysis in CCE

**FIGURE 1:**
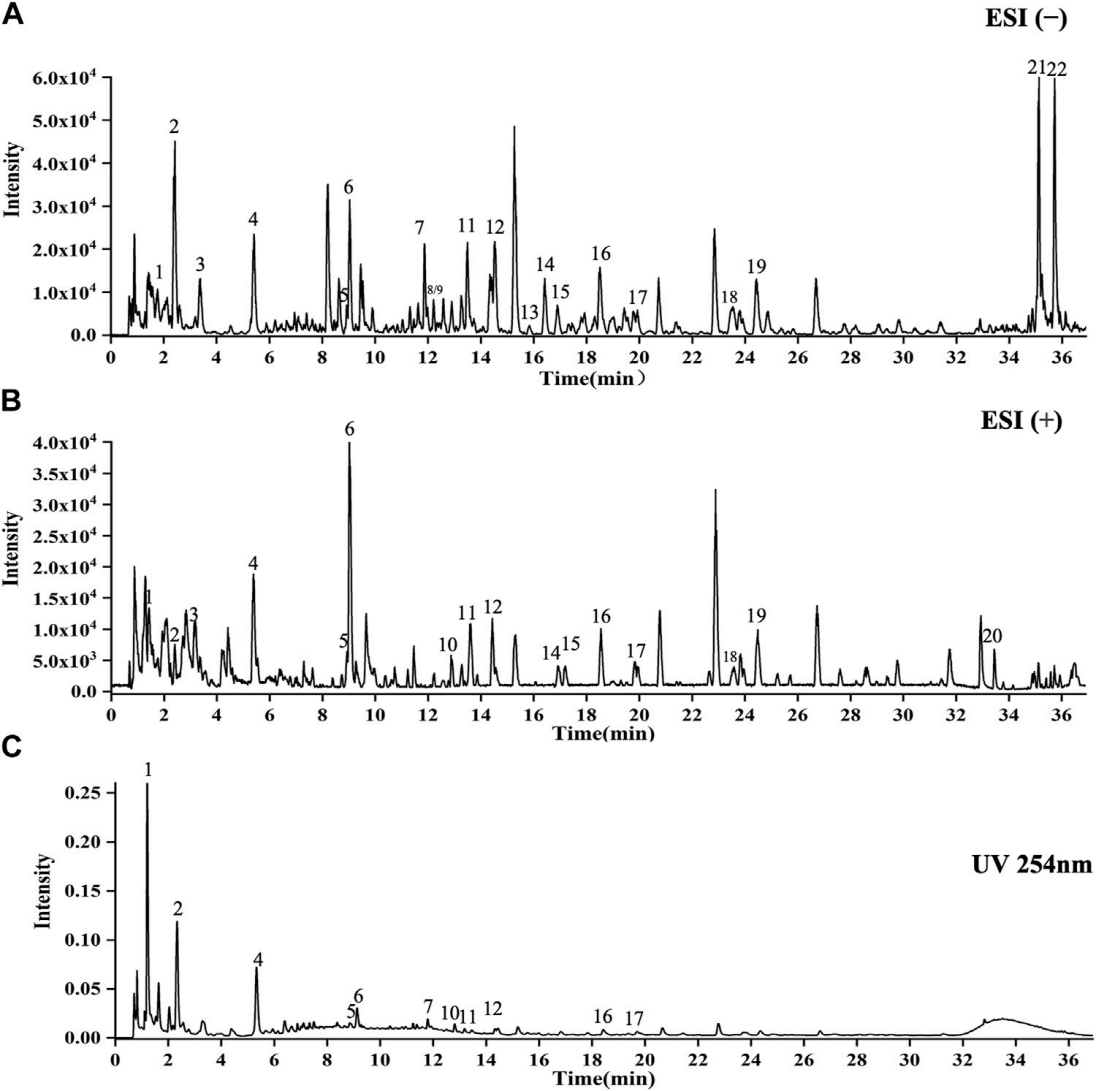
UPLC-Q-TOF-MS analysis of CCE. **(A)** ESI(−) mode. **(B)** ESI(+) mode. **(C)** UV254 nm mode. *X*-axis indicates when the peaks appear, and *y*-axis shows the intensities of the peaks.

As shown in Figure 1, 22 compounds (1. N-Fructosyl pyroglutamate, 2. Uridine, 3. Cyclic guanosine monophosphate Guanosine, 4. Guanosine, 5. L-tryptophan, 6. Phenylalanylalanine, 7. 3-Methoxy-L-tyrosine, 8. Sinapoylglucose, 9. Komaroveside A or isomer, 10. Quercetin 3-sophoroside 7-glucoside, 11. Komaroveside C, 12. Kaempferol 3-sophoroside 7-glucoside, 13. Quercetin-3-O-sophoroside, 14. Corchoionoside A, 15. Sinapic acid, 16. Kaempferol-3-O-sophoroside, 17. Hyperoside, 18. Naringenin-7-O-glucoside, 19. Astragalin, 20. 1,2,2′-Trisinapoylgentiobioside, 21. Trihydroxyoctadecadienoic acid, and 22. Trihydroxyoctadecenoic acid.) were identified as part of the study and are presented in Supplementary Materil TS4.

### 3.2 Body weights

**FIGURE 2:**
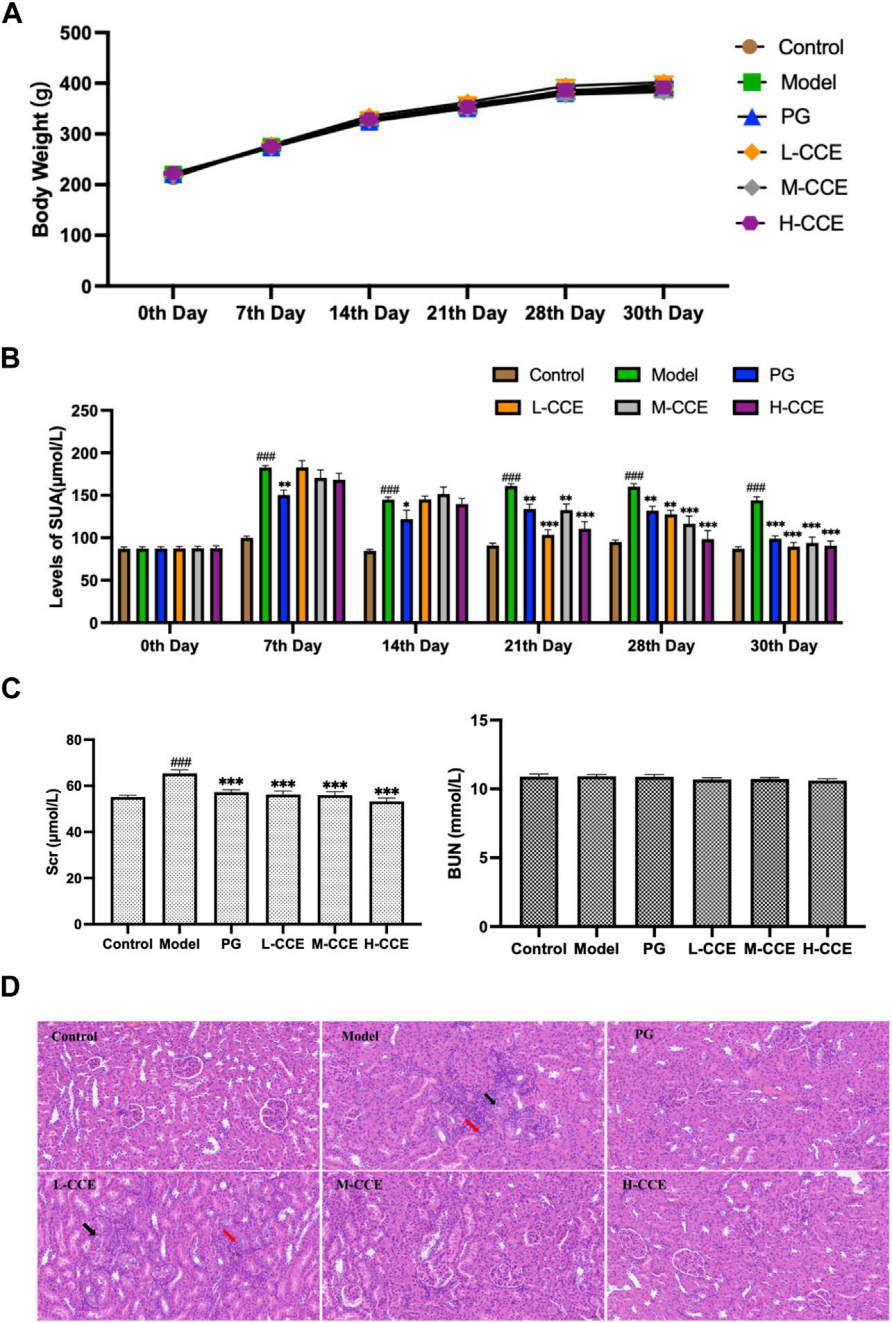
Anti-hyperuricemic Effect and Histopathological Analysis. **(A)** Effects of CCE on body weight. **(B)** Effects of CCE on levels of UA. Data are expressed as means ± SEM, *n* = 8. Compared with Control group: ^##^ *p* < 0.01, ^###^ *p* < 0.001; Compared with Model group: **p < 0.01, ***p < 0.001. **(C)** Effects of CCE on levels of Scr and BUN. Data are expressed as means ± SEM, *n* = 8. Compared with Control group: ^###^ *p* < 0.001; compared with Model group: ***p < 0.001. **(D)** Histopathological observation of renal tissue (20×).

As can be seen in Figure 2A, no significant changes in body weight of the PO-induced hyperuricemic animal model were present. Furthermore, intervention with CCE did not result in any noticeable changes in body weight in rats.

### 3.3 Levels of UA

As shown in Figure 2B, the UA levels in the Model group were significantly higher from the 7th day to the 30th day. The PG group exhibited considerably lower levels of UA compared to the Model group from the 7th to the 30th day, which validates the successful induction of hyperuricemia in the Model group. In comparison to the Model group, the L-CCE group showed a tendency towards lower UA levels from the 21st to the 30th day, and the H-CCE group demonstrated a significant reduction in UA levels from the 21st to the 30th day.

### 3.4 Levels of Scr and BUN

In Figure 2C, the results illustrate that levels of Scr were significantly increased in the Model group. After treatment with L-, M-, and H-CCE, a significant reduction in Scr levels was observed. These reductions were similar to those observed in the PG group. However, the treatments did not show a significant difference in BUN levels. These findings indicate that the CCE had a remarkable protective effect on the kidney.

### 3.5 Histopathological analysis

Figure 2D demonstrates the histopathological analysis of renal structure in different groups. In the Control group, the renal structure appeared normal, with a well-defined outline. The epithelial cells were rounded and intact, with a well-organized and regular arrangement of the brush border and no apparent abnormalities in the medulla. The connective tissue between the urinary tubules was interstitial, and there was no apparent hyperplasia in the interstitium and no noticeable inflammatory changes. In contrast, a small amount of tubular atrophy (indicated by the black arrows) and lymphocytic infiltration (indicated by the red arrows) was seen locally in the renal cortex in the Model group. However, the supplementation of M-CCE and H-CCE significantly alleviated the inflammatory changes in the renal histopathology, indicating that CCE had an excellent protective effect against hyperuricemic-induced inflammatory changes.

### 3.6 Metabolomic analysis

#### 3.6.1 Renal metabolomics analysis

UPLC-Q-TOF/MS was employed to collect the data of renal samples, and 5,433 metabolites were detected in positive ion modes (ESI(+)) and 7,553 metabolites in negative ion modes (ESI(−)).

**FIGURE 3:**
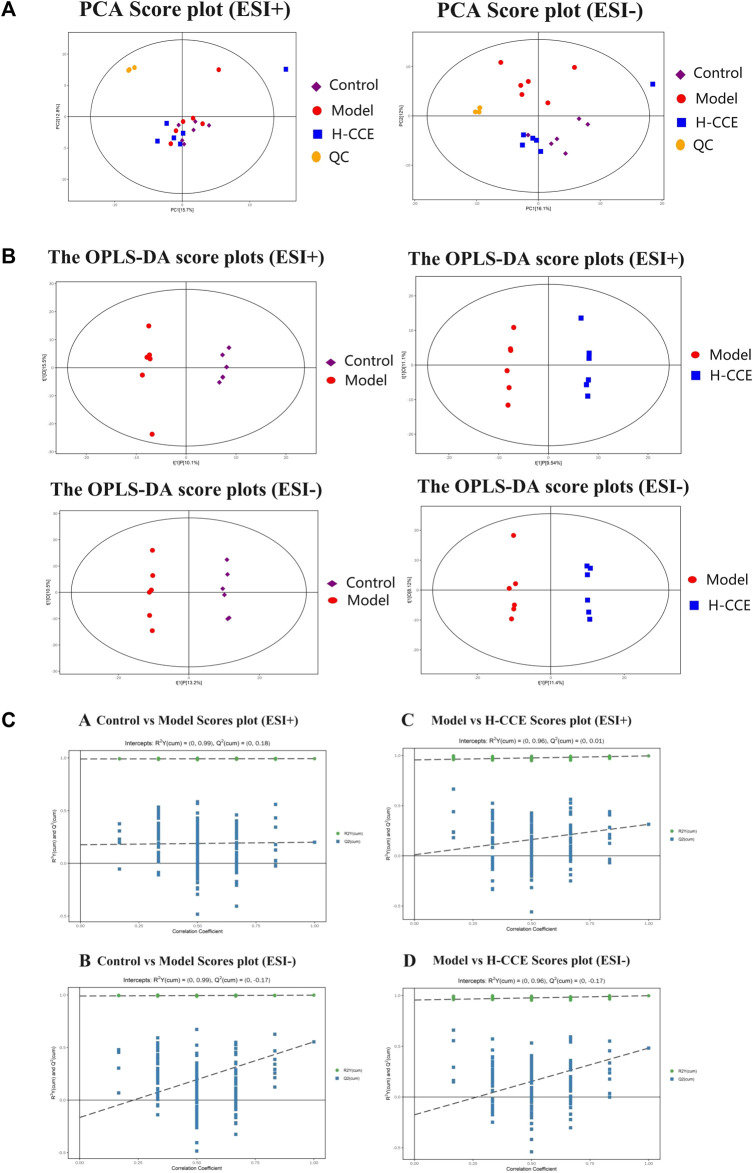
Metabolomic Analysis. **(A)** Score scatter plot for PCA model with QC. **(B)** OPLS-DA score plots. **(C)** Permutation tests. The Control group is shown with purple squares, the Model group with red dots, the CCE group with blue squares, and the QC samples with orange dots. All samples are in ESI(+) mode and ESI(−) modes separately.

The stability and repeatability of the analytical methods and instruments were assessed using QC samples in the metabolomics analysis. The QC samples demonstrated excellent reproducibility in terms of peak response intensity and retention time, indicating the favorable stability of the instruments throughout the analysis process. Principal component analysis (PCA), an unsupervised pattern recognition analysis method, was employed to visualize the trends among the groups. As depicted in Figure 3A, the QC samples were closely clustered in the score plot for both ESI(+) and ESI(−) modes, indicating the system’s stability and repeatability, thus ensuring the reliability of the experiment. Subsequently, the PCA score plot was used to distinguish the differences between the Control group, Model group, and H-CCE group. The PCA analysis revealed a distinct separation of renal metabolite profiles between the Model group and the other groups in the ESI(+) mode. In the ESI(−) mode, the Model group showed a clear separation trend from both the Control group and the H-CCE group. These findings suggest that hyperuricemia induces metabolic alterations in renal tissue and perturbs the endogenous small molecule metabolites.

Supervised OPLS-DA was performed to further differentiate the metabolic profiles between the groups in ESI(+) and ESI(−) modes, respectively. This analysis identified potential biomarkers that contribute to the observed variations (Figure 3B). The results demonstrated significant separation between the Control group and the Model group (ESI(+): R2Y (cum)=(0, 0.99), Q2 (cum)=(0, 0.18); ESI(−): R2Y (cum)=(0, 0.99), Q2 (cum)=(0, −0.17)). Additionally, the H-CCE group exhibited significant separation from the Model group (ESI(+): R2Y (cum)=(0, 0.96), Q2 (cum)=(0, 0.01); ESI(−): R2Y (cum)=(0, 0.96), Q2 (cum)=(0, −0.17). The sample of each group can be completely separated in the PC1 dimension, indicating distinct characteristics in the renal metabolomics of rats in each group.

To validate the reliability of the OPLS-DA mode and ensure that it was not overfitted, 200 displacement tests were performed. The results, as depicted in Figure 3C, showed that all R2 and Q2 values in the tests were lower than the original values. Additionally, the Q2 value approached zero, indicating that the OPLS-DA mode did not exhibit overfitting. These findings suggest that the results obtained from the OPLS-DA analysis are highly reliable.

#### 3.6.2 Differential metabolites and pathway enrichment

**FIGURE 4:**
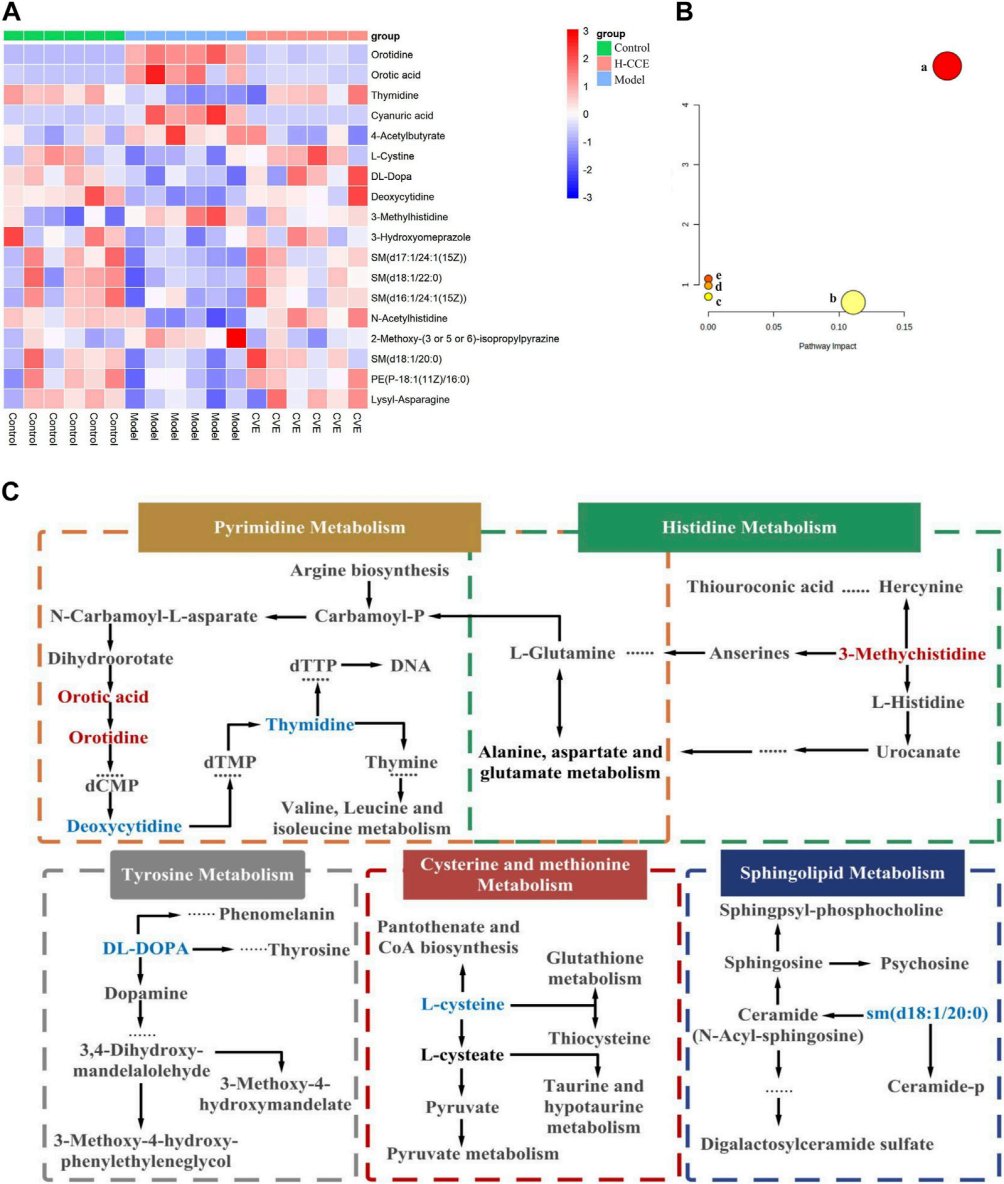
Differential Metabolites and pathway analysis. **(A)** Cluster heat map. **(B)** Metabolic pathway analysis. **(C)** Metabolic pathways of biomarkers. Metabolites with red markers show a significant increase in the Model group compared to the Control group but a decrease in the CCE group compared with the Model group. Metabolites with blue features indicate a reduction in the Model group compared to the Control group but an increase in the CCE group compared with the Model group.

By comparing the obtained metabolites between the Control and Model groups with the online METLIN and HMDB databases, a total of 49 different metabolites were identified, which could be considered biomarkers of hyperuricemia induction. The metabolites identified were found to differ between the Model and CCE groups. Among the 49 biomarkers identified between the Control and Model groups, 18 metabolites showed alterations after CCE administration, of which 11 were in the ESI(+) mode, and 7 were in the ESI(−) mode. The results are presented in Table 1, and a heatmap is shown in Figure 4A. These 18 differential metabolites were subjected to signaling pathway enrichment. The results, displayed in Figures 4B, C, revealed the main 5 pathways, which were pyrimidine metabolism, tyrosine metabolism, cysteine and methionine metabolism, sphingolipid metabolism, and histidine metabolism pathways.

**TABLE 1:** Differential metabolites and their change trends.

No.	Differential metabolites	Mode	Model vs control				Model vs CCE			
			VIP	Ratio	Trend	Significant	VIP	Ratio	Trend	Significant
**1**	Orotidine	ESI(−)	6.60	49.98	**↑**	***	5.12	9.23	**↓**	***
**2**	Orotic acid	ESI(−)	4.71	11.13	**↑**	**	4.03	6.00	**↓**	*
**3**	Thymidine	ESI(−)	1.80	0.72	**↓**	***	1.32	0.78	**↑**	*
**4**	Cyanuric acid	ESI(−)	6.65	72.99	↑	**	6.48	44.09	**↓**	**
**5**	4-Acetylbutyrate	ESI(−)	1.84	1.51	**↑**	**	1.85	1.49	**↓**	*
**6**	L-Cystine	ESI(−)	1.52	0.71	**↓**	*	1.95	0.64	**↑**	**
**7**	DL-Dopa	ESI(−)	1.56	0.75	**↓**	**	1.58	0.72	**↑**	*
**8**	Deoxycytidine	ESI(+)	1.93	0.77	**↓**	***	1.59	0.81	**↑**	**
**9**	3-Methylhistidine	ESI(+)	3.33	2.30	**↑**	**	1.65	1.40	**↓**	*
**10**	3-Hydroxyomeprazole	ESI(+)	1.48	0.81	**↓**	*	1.23	0.85	**↑**	*
**11**	SM(d17:1/24:1 (15Z))	ESI(+)	2.52	0.60	**↓**	**	2.32	0.63	**↑**	**
**12**	SM(d18:1/22:0)	ESI(+)	2.03	0.67	**↓**	*	2.11	0.68	**↑**	**
**13**	SM(d16:1/24:1 (15Z))	ESI(+)	2.07	0.67	**↓**	*	1.95	0.68	**↑**	*
**14**	N-Acetylhistidine	ESI(+)	1.95	0.73	**↓**	**	2.07	0.71	**↑**	**
**15**	2-Methoxy-(3 or 5 or 6)-isopropylpyrazine	ESI(+)	2.28	1.59	**↑**	*	2.29	1.65	**↓**	*
**16**	SM(d18:1/20:0)	ESI(+)	1.70	0.76	**↓**	*	1.81	0.72	**↑**	*
**17**	PE (P-18:1 (11Z)/16:0)	ESI(+)	2.20	0.62	**↓**	*	2.33	0.62	**↑**	*
**18**	Lysyl-Asparagine	ESI(+)	1.88	0.74	**↓**	**	1.68	0.73	**↑**	*

*p < 0.05,

**p < 0.01,

***p < 0.001. The arrows (↑ and ↓) represent the increase or decrease of the biomarkers between groups.

### 3.7 Network pharmacology analysis

#### 3.7.1 Target prediction and construction of the CCE-compound–hyperuricemia-target network

**FIGURE 5:**
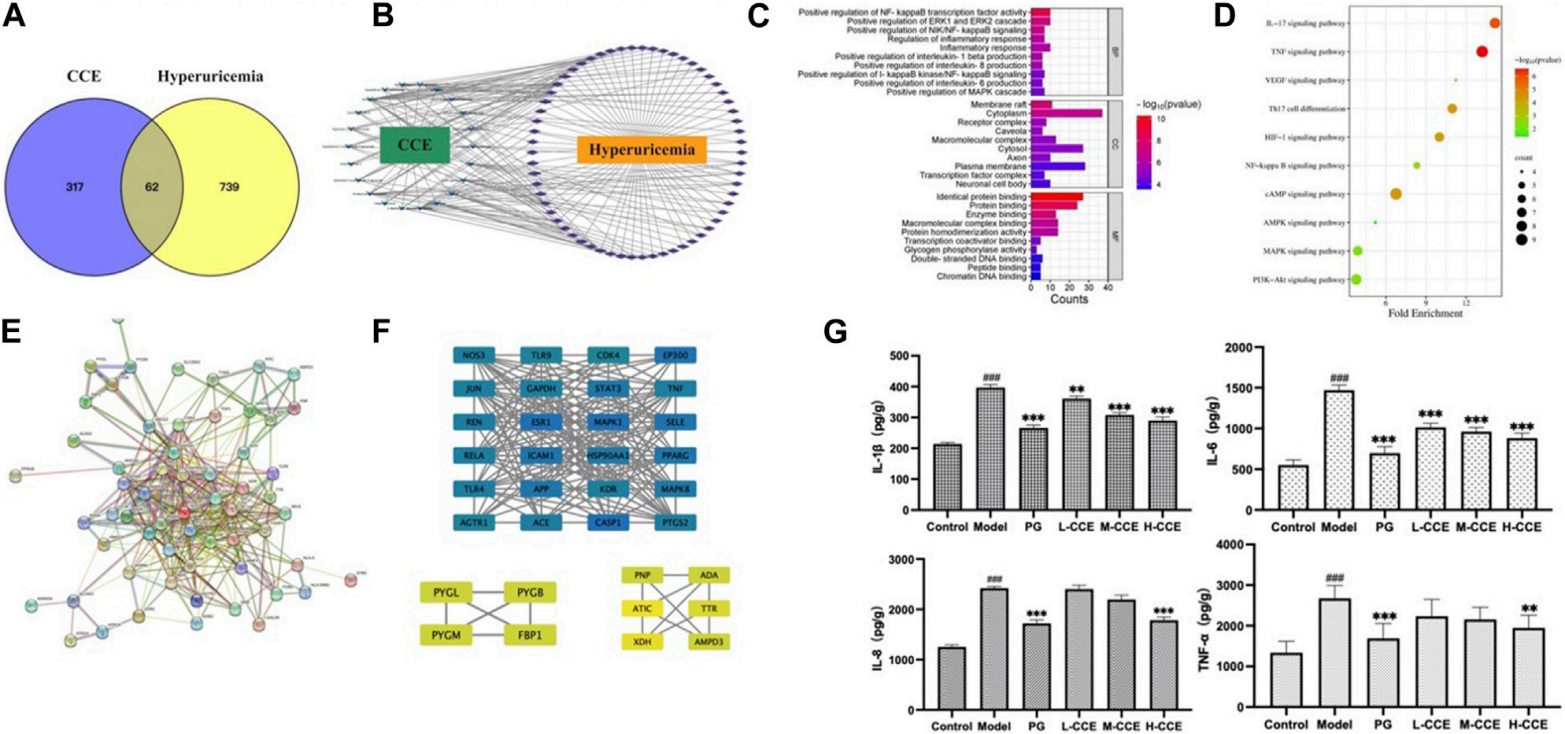
Target network analysis. **(A)** Venn diagram; **(B)** CCE-compound–target–hyperuricemia network; blue V shapes indicate compounds, purple rhombuses indicate targets, the number of connections of a node is defined as the degree (edges), which is proportional to the importance of the node; **(C)** bar chart of GO analysis; **(D)** bubble chart of KEGG pathways; **(E)** PPI network analysis; **(F)** map of the sub-network screened from PPI by MCODE, the rectangles vary in color from blue to green to yellow, representing the degree from high to low. **(G)** Effects of CCE on levels of inflammatory factors on hyperuricemic rats. Data are expressed as means ± SEM, *n* = 8. Compared with Control group: ###*p* < 0.001; compared with Model group: **p < 0.01, ***p < 0.001.

UPLC-Q-TOF-MS was used to obtain the prototypes of CCE, resulting in the identification of 379 targets. Additionally, 801 hyperuricemia-related markers were obtained. Then, the 62 overlapping targets were selected using the Venn tool (Figure 5A). Subsequently, a CCE-compound-hyperuricemia-target network was constructed, incorporating 22 compounds and 62 targets, with 86 nodes and 213 edges (Figure 5B).

#### 3.7.2 Functional enrichment and pathway analysis

We next performed the gene ontology (GO) functional enrichment analysis. 259 BP terms, 34 CC terms, and 48 MF terms were found significantly enriched. Figure 5C shows that the top 10 GO analyses in BP revealed target abundance in the regulation of inflammatory factors, such as NF-κB, IL-1β, IL-6, and IL-8. In CC, targets were accumulated in the cytoplasm and plasma membranes, while in MF, targets were primarily responsible for identical protein binding, protein binding, and enzyme binding. Furthermore, 83 KEGG pathways were enriched. The 10 key pathways are shown in Figure 5D, including the IL-17 signaling pathway and NF-κB signaling pathway.

#### 3.7.3 PPI network and module analysis

As depicted in Figure 5E, the PPI (Protein-Protein Interaction) network has been constructed, and the MCODE (Molecular Complex Detection) plugin was used to identify the sub-networks. We also performed modular analysis and identified 34 hub genes through cluster analysis, as shown in Figure 5F.

#### 3.7.4 Levels of inflammatory cytokines validation

We then conducted ELISA tests on the inflammatory cytokines in the renal tissues to validate the prediction based on the GO analyses. As shown in Figure 5G, there was an increase in the levels of IL-1β and IL-6 in the Model group, while L-, M-, and H-CCE treatments were found to decrease IL-1β and IL-6, reaching a similar level as the PG group. Additionally, IL-8 levels increased in the Model group, while H-CCE treatment reduced it to a level comparable to that of the PG group. Furthermore, TNF-α showed a similar trend, with elevated levels in the Model group, which was able to be decreased by H-CCE to the levels in the PG group.

### 3.8 Prediction of crucial gene targets

**FIGURE 6:**
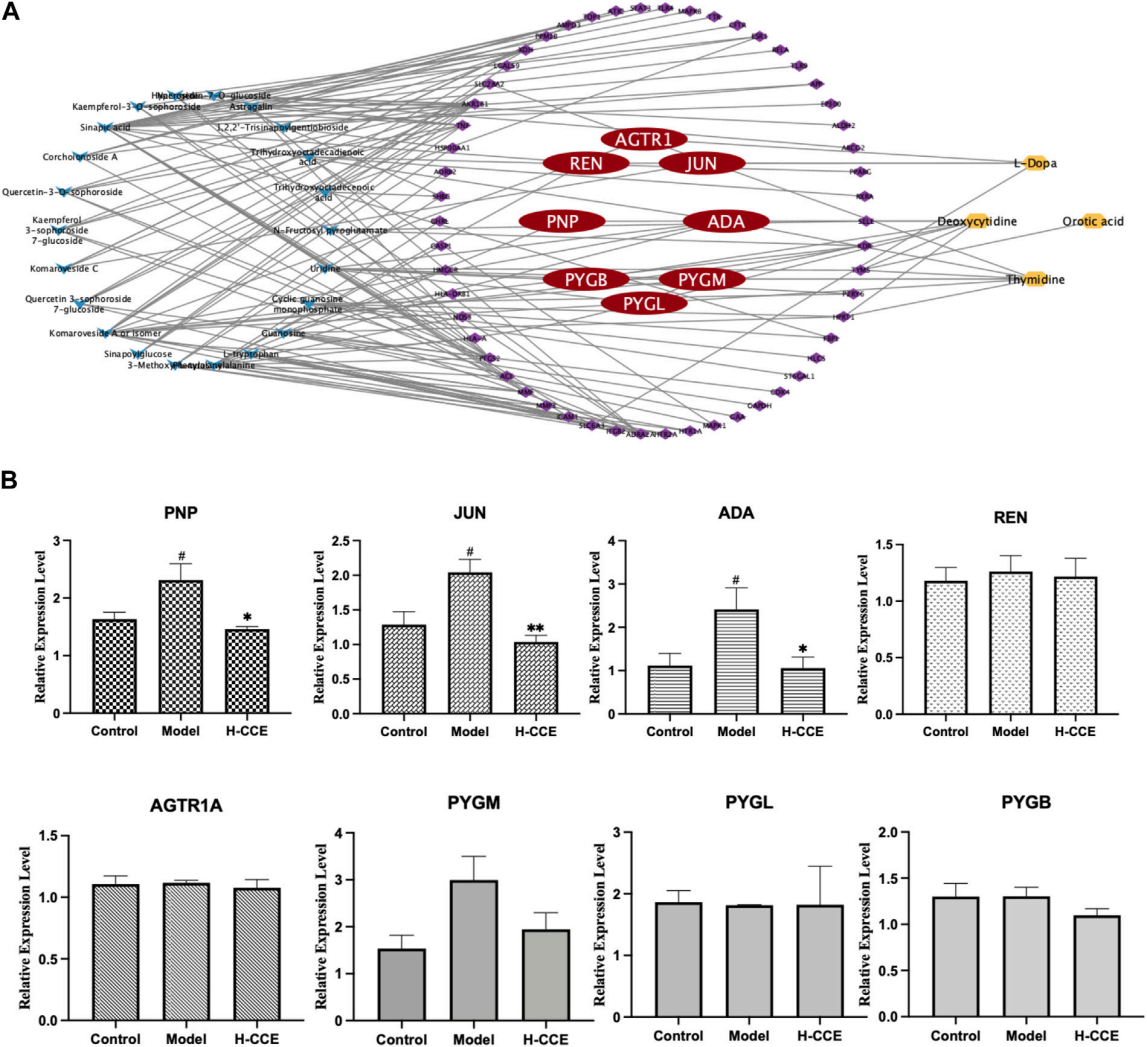
Prediction of key gene targets and RT-qPCR validation. **(A)** Compound–target–metabolite network of CCE. The blue diamonds represent CCE; the purple and red round rectangles represent the targets and key gene targets, and the yellow ellipses represent differential metabolites. **(B)** Related mRNA expression levels of the key gene targets. Data are expressed as means ± SEM, *n* = 3. Compared with Control group: ^#^ *p* < 0.05; compared with Model group: *p < 0.05, **p < 0.01, ***p < 0.001.

Eighteen differential metabolites and 62 targets were imported into MetaboAnalyst to construct the gene-metabolite network (see Supplementary Material FS1 for details); the resulting network contained 12 targets and 4 differential metabolites. The Compound–Target–Metabolite network was then constructed as shown in Figure 6A; 8 key gene targets (AGTR1, JUN, REN, ADA, PNP, PYGM, PYGL, and PYGB) were predicted and further verified by RT-qPCR.

### 3.9 RT-qPCR analysis

As shown in Figure 6B, the Model group exhibited significantly elevated mRNA expression levels of ADA, PNP, and JUN compared to the Control group. However, treatment with H-CCE demonstrated a notable reversal of these key gene targets.

## 4 Discussion

This study employed a comprehensive approach to investigate the anti-hyperuricemic mechanism of CC using the biochemical test, histopathological assay, and metabonomics analysis. Furthermore, metabolomics was integrated with network pharmacology for the prediction of critical gene targets, followed by RT-qPCR to reveal the mechanism. By utilizing this multifaceted approach, this study offers a more in-depth understanding of CC, a valuable and highly anticipated traditional herbal medicine with Se hyperaccumulating characteristics, to explore the potential pharmacological mechanisms of action underlying its anti-hyperuricemic effects.

### 4.1 Anti-hyperuricemic effect of CCE associated with anti-inflammatory activity

The liver is primarily responsible for synthesizing urate, two-thirds of which is excreted from the body through the kidneys (Yanai et al., 2021). Scr and BUN levels are essential biochemical indicators for detecting any abnormalities in renal function (Xu et al., 2021). In this study, it was demonstrated that CCE reduced serum UA and Scr levels in hyperuricemic rats. Additionally, the results of the renal histopathological examination showed that PO induced a small amount of lymphocyte infiltration localized in the renal cortex, indicating inflammation caused by urate deposition. However, CCE treatment reversed this inflammation and improved the lymphocyte infiltration, which further suggests an anti-inflammatory mechanism underlying the beneficial effects of CCE in ameliorating hyperuricemia.

Specifically, inflammation plays a significant role in developing renal injury caused by urate. Soluble urate activates NLRP3 inflammatory vesicles, inducing the release of IL-1β (Zhou et al., 2011) and triggering the inflammatory response. Urate also causes endothelial damage and vascular dysfunction, resulting in the release of IL-6, IL-8, and TNF-α (Zhen and Gui, 2017), which further exacerbates renal injury. The findings from this study confirmed that CCE reversed the elevation of IL-1β, IL-6, IL-8, and TNF-α in the kidneys of hyperuricemic rats, which suggests that CCE can alleviate the state of endothelial injury and vascular dysfunction of the kidneys caused by urate and contribute to the reduction of UA levels.

Based on the findings in the network pharmacology section, it can be seen that the components responsible for the UA-lowering effects and improvement of inflammatory conditions of CCE are likely to be flavonoids, such as hyperoside and astragalin. However, the UPLC-Q-TOF-MS study revealed that the levels of these two compounds are very low, and no further investigations into these individual compounds were conducted in this study. However, it would be worthwhile to conduct further in-depth research on them.

### 4.2 Screening and identification of differential metabolites and signaling pathways in renal tissues by metabolomics analysis for CCE anti-hyperuricemia effects

The results from metabolomic analysis demonstrated that the anti-hyperuricemic effects of CCE are primarily associated with alterations in pyrimidine, tyrosine, cysteine and methionine, sphingolipid, and histidine metabolism.

UA, the end product of purine metabolism (Choi et al., 2023), is derived from the breakdown of purines through a series of catalytic reactions (Saito et al., 2021). Pyrimidine metabolism involving orotic acid and orotidine was the first core of the hyperuricemia metabolic networks. In this study, the content of orotidine and orotic acid in the renal tissues of the Model group was found to increase significantly but decrease in the H-CCE group. Conversely, the content of thymidine and deoxycytidine decreased significantly in the Model group but increased in the H-CCE group. These findings suggest that CCE exerts its UA-lowering effects by influencing pyrimidine metabolism. In addition, amino acid metabolism plays a crucial role in regulating protein and energy metabolism. Since abnormal amino acid metabolism can also cause metabolic disturbances, the metabolism of tyrosine, which is a type of amino acid, is particularly important.

The increase in tyrosine levels stimulated by monosodium urate (MSU) and activation of neutrophils and tyrosine kinase Tec can induce the release of IL-1β, IL-8, IL-1, and tyrosine (Popa-Nita et al., 2008), which triggers an inflammatory response. The results suggest that CCE can modulate tyrosine metabolism in the metabolic pathway of hyperuricemia to reduce the inflammatory response. Furthermore, histidine metabolism (Stifel and Herman, 1971) is also closely related to inflammation. It can regulate NLRP6 inflammasome, leading to downstream secretion of antimicrobial peptides and promote the secretion of interleukin-18 (IL-18). CCE can impact histidine metabolism in the metabolic pathway of hyperuricemia, thereby improving the inflammatory state caused by urate.

Sphingolipids are a class of amphiphilic lipids that consist of a sphingolipid backbone and phospholipids, such as ceramide (Cer), sphingomyelin (SM), and gangliosides (Ogretmen, 2018). In this study, it was found that CCE reversed the decrease in the concentration of DL-DOPA in tyrosine metabolism, 3-Methylhistidine in histidine metabolism, and sm (d18:1/20:0) in sphingolipid metabolism caused by hyperuricemia. Furthermore, the elevated cystine levels (the disulphide form of cysteine) were positively associated with oxidative stress (Chiang et al., 2022) and endothelial dysfunction (Keller et al., 2019). CCE reversed the decreased level of L-cystine, which shows the protective effect of CCE against hyperuricemia-induced vascular endothelial dysfunction and oxidative stress.

Metabolomic analyses conducted on renal tissues further demonstrated that CCE may ameliorate the urate-induced inflammatory status by modulating disturbed metabolic pathways.

### 4.3 Biological assessment of network pharmacology-integrated metabolomics analysis combined with anti-hyperuricemic effect of CCE associated with anti-inflammatory activity via regulates ADA, PNP, and JUN mRNA expression in the renal tissues

A combination of network pharmacology and metabolomics was employed to analyze and predict key gene targets so that specific metabolites could be linked to drug targets to identify key gene targets and reveal the underlying mechanisms of action of CCE in its anti-hyperuricemic effect. The results showed that AGTR1, JUN, REN, ADA, PNP, PYGM, PYGL, and PYGB were the key gene targets for the anti-hyperuricemic effect of CCE. Further research confirmed that CCE has the potential to regulate the mRNA expression of ADA, PNP, and JUN in the kidney, thereby alleviating hyperuricemia associated with anti-inflammatory activity.

Adenosine deaminase (ADA) is an essential inflammatory enzyme involved in the formation of adenosine UA (Moustafa et al., 2019). One of its critical catalytic enzymes is inosine, which is phosphorylated by purine nucleoside phosphorylase (PNP) to produce α-D-deoxyribose-1-phosphate and hypoxanthine (Tumova et al., 2021). This product plays a crucial role in purine metabolism (Fekrvand et al., 2019), thus in turn affecting UA metabolism. The results indicated that CCE inhibited mRNA expression levels of ADA and PNP in the kidney and reduced UA levels. Interestingly, our study also showed that CCE did not affect xanthine oxidase (XOD) activity in serum (see Supplementary Materil TS5 for details). This finding suggests that the process by which CCE influences UA metabolism may be more closely associated with the adjustment of inflammatory states, which is consistent with the results showing that CCE can reduce the expression of inflammatory factors. In future studies, we will conduct experiments to examine XOD activity in the kidney and liver in order to further investigate this interesting finding and elucidate the underlying mechanism. It suggests that the mechanism by which CCE ameliorates the inflammatory condition in hyperuricemia is closely related to its inhibitory effect on ADA inflammatory enzymes.

Meanwhile, the FOS and JUN families are part of the AP-1 pathway, which can regulate inflammatory responses by influencing angiogenesis and invasion (Schonthaler et al., 2011). Originally located in the cytosol, c-Fos and c-Jun translocate into the nucleus and dimerize after they are activated by their upstream kinases, mitogen-activated protein kinases (MAPKs) (Whitmarsh and Davis, 1996; Schonthaler et al., 2011). MAPKs comprise a family of serine/threonine protein kinases, notably including extracellular signal-regulated kinase (ERK), c-Jun N-terminal kinase (JNK), and p38 (Whitmarsh and Davis, 1996). JNK, c-Jun, and p38 (Silvers et al., 2003) can be activated to modulate inflammatory responses. The results suggest that CCE improves the inflammatory state associated with hyperuricemia by inhibiting the expression of JUN mRNA in the AP-1 signaling pathway. Therefore, our results proved that ADA, PNP, and JUN are the gene targets of CCE and that their modulation contributes to the anti-hyperuricemic effect associated with the anti-inflammatory activity of CCE. However, it is important to note that protein level assays were not performed in this study due to the specific methodology employed, which combined metabolomics with network pharmacological analysis for gene target prediction, and included validation at the gene expression level. Future studies should focus on investigating the protein level mechanisms in order to shed light on the modulation of the anti-hyperuricemic effect associated with the anti-inflammatory activity of CCE.

Overall, the results of the study suggest that, despite limited data, it is feasible to employ the metabolomic approach in conjunction with network pharmacology to predict gene targets that are key to the UA-lowering effects of CCE. Further analyses confirmed a correlation between the ability of CCE to reduce UA levels and the improvement of the inflammatory state of the kidneys. This approach is based on changes in metabolite levels and incorporates targets predicted by network pharmacology to find gene targets of action, enabling predictive analyses of gene targets at the metabolite level. Therefore, this approach helps to advance the overall understanding of the mechanism of action of herbal aqueous extracts. In addition, for in-depth elucidation of the mechanism of action at the protein level, future studies could consider incorporating proteomics as well as transcriptomics based on target proteins, differentially expressed genes, and the final validation of the key targets.

In conclusion, the results indicated that CCE exhibits a significant anti-hyperuricemia effect, and the mechanism of action is associated with its anti-inflammatory activity by reversing the elevation of renal inflammatory cytokines, restoring the disordered metabolic pathways, and inhibiting the expression of ADA, PNP, and JUN mRNA in renal tissues.

## Data Availability

The datasets presented in this study can be found in online repositories. The names of the repository/repositories and accession number(s) can be found in the article/Supplementary Material.
