# Supplementary material for: Integrative metabolomic and network pharmacological analysis reveals potential mechanisms of Cardamine circaeoides Hook.f. & Thomson in alleviating potassium oxonate-induced asymptomatic hyperuricemia in rats
Source: Front Pharmacol. 2023 Nov 2;14:1281411. doi: 10.3389/fphar.2023.1281411 (PMC10652788; doi:10.3389/fphar.2023.1281411)
Supplement: Supplementary file 1 [file Table1.DOCX]

**Table 1. Differential metabolites and their change trends.**

| **No.** | **Differential metabolites** | | **Mode** | | **Model vs Control** | | | | | |  | | **Model vs CCE** | | | | | |  | |
| --- | --- | --- | --- | --- | --- | --- | --- | --- | --- | --- | --- | --- | --- | --- | --- | --- | --- | --- | --- | --- |
|  |  |  |  |  | **VIP** | | **Ratio** | | **Trend** | | **Significant** | | **VIP** | | **Ratio** | | **Trend** | | **Significant** |  |
| **1** | Orotidine | | ESI(−) | | 6.60 | | 49.98 | |  | | *** | | 5.12 | | 9.23 | |  | | *** | |
| **2** | Orotic acid | | ESI(−) | | 4.71 | | 11.13 | |  | | ** | | 4.03 | | 6.00 | |  | | * | |
| **3** | Thymidine | | ESI(−) | | 1.80 | | 0.72 | |  | | *** | | 1.32 | | 0.78 | |  | | * | |
| **4** | Cyanuric acid | ESI(−) | | 6.65 | | 72.99 | |  | | ** | | 6.48 | | 44.09 | |  | | ** | |  |
| **5** | 4-Acetylbutyrate | | ESI(−) | | 1.84 | | 1.51 | |  | | ** | | 1.85 | | 1.49 | |  | | * | |
| **6** | L-Cystine | | ESI(−) | | 1.52 | | 0.71 | |  | | * | | 1.95 | | 0.64 | |  | | ** | |
| **7** | DL-Dopa | | ESI(−) | | 1.56 | | 0.75 | |  | | ** | | 1.58 | | 0.72 | |  | | * | |
| **8** | Deoxycytidine | | ESI(+) | | 1.93 | | 0.77 | |  | | *** | | 1.59 | | 0.81 | |  | | ** | |
| **9** | 3-Methylhistidine | | ESI(+) | | 3.33 | | 2.30 | |  | | ** | | 1.65 | | 1.40 | |  | | * | |
| **10** | 3-Hydroxyomeprazole | | ESI(+) | | 1.48 | | 0.81 | |  | | * | | 1.23 | | 0.85 | |  | | * | |
| **11** | SM(d17:1/24:1(15Z)) | | ESI(+) | | 2.52 | | 0.60 | |  | | ** | | 2.32 | | 0.63 | |  | | ** | |
| **12** | SM(d18:1/22:0) | | ESI(+) | | 2.03 | | 0.67 | |  | | * | | 2.11 | | 0.68 | |  | | ** | |
| **13** | SM(d16:1/24:1(15Z)) | | ESI(+) | | 2.07 | | 0.67 | |  | | * | | 1.95 | | 0.68 | |  | | * | |
| **14** | N-Acetylhistidine | | ESI(+) | | 1.95 | | 0.73 | |  | | ** | | 2.07 | | 0.71 | |  | | ** | |
| **15** | 2-Methoxy-(3 or 5 or 6)-isopropylpyrazine | | ESI(+) | | 2.28 | | 1.59 | |  | | * | | 2.29 | | 1.65 | |  | | * | |
| **16** | SM(d18:1/20:0) | | ESI(+) | | 1.70 | | 0.76 | |  | | * | | 1.81 | | 0.72 | |  | | * | |
| **17** | PE(P-18:1(11Z)/16:0) | | ESI(+) | | 2.20 | | 0.62 | |  | | * | | 2.33 | | 0.62 | |  | | * | |
| **18** | Lysyl-Asparagine | | ESI(+) | | 1.88 | | 0.74 | |  | | ** | | 1.68 | | 0.73 | |  | | * | |

^*^ *p* < 0.05, ^**^ *p* < 0.01, ^***^ *p* < 0.001. The arrows (↑ and ↓) represent the increase or decrease of the biomarkers between groups.
