# Supplementary material for: Integrative metabolomic and network pharmacological analysis reveals potential mechanisms of Cardamine circaeoides Hook.f. & Thomson in alleviating potassium oxonate-induced asymptomatic hyperuricemia in rats
Source: Front Pharmacol. 2023 Nov 2;14:1281411. doi: 10.3389/fphar.2023.1281411 (PMC10652788; doi:10.3389/fphar.2023.1281411)

**Supplementary Data**

**Table S1. Mobile Phase Gradient of UPLC-Q-TOF-MS**

**Table S2. Mass parameters (Sciex Triple TOF 4600 LC-MS)**

**Table S3. Primers used for quantitative RT-qPCR**

**Table S4. List of the compounds identified**

**Table S5. Effects of CCE on levels of XOD on hyperuricemic rats**

**Figure S1. Gene–Metabolite networks by MetaboAnalyst.**

**Table S1. Mobile Phase Gradient of UPLC-Q-TOF-MS**

| Time (min) | A% | B% |
| --- | --- | --- |
| 0～3 | 0 | 100 |
| 3～10 | 0～12 | 100～88 |
| 10～13 | 12 | 88 |
| 13～30 | 12～20 | 88～80 |
| 30～35 | 20～45 | 80～55 |
| 35～40 | 45～95 | 55～5 |
| 40～42 | 95 | 5 |
| 42～42.1 | 95～0 | 5～100 |
| 42.1～45 | 0 | 100 |

Note: The UPLC instruments were Waters H-Class (Waters Technologies, USA) and AB Sciex Triple TOF® 4600 LC/MS (SCIEX, USA) and Waters CORTECS® UPLC® T3 (2.1 × 100 mm, 1.6 µm). The mobile phase contains acetonitrile (A) and 0.1% aqueous formic acid (B).

**Table S2. Mass parameters (Sciex Triple TOF 4600 LC-MS)**

| MS |  | MS/MS | Data |
| --- | --- | --- | --- |
| TOF mass range | 50～1700 | MS/MS mass range | 50～1250 |
| Ion Source Gas 1（psi） | 50 | Declustering Potential（V） | 100 |
| Ion Source Gas 2（psi） | 50 | Collision Energy（eV） | ±40 |
| Curtain Gas（psi） | 35 | Collision Energy Spread（eV） | 20 |
| Ion Spray Voltage Floating (V) | -4500/5000 | Ion Release Delay（ms） | 30 |
| Ion Source Temperature (°C) | 500 | Ion Release Width（ms） | 15 |
| Declustering Potential（V） | 100 |  |  |
| Collision Energy（eV） | 10 |  |  |

**Table S3. Primers used for quantitative RT-qPCR.**

| **Genes** | **Forward (5'–3' )** | **Reverse (5'–3' )** |
| --- | --- | --- |
| GAPDH  AGTR1 | CTGGAGAAACCTGCCAAGTATG  ACAACTGCCTGAACCCTCTG | GGTGGAAGAATGGGAGTTGCT  AAGGCCGGTAAGAAAGCGTG |
| JUN | GAACCGCATCGCTGCCT | CATGAGTTGGCACCCACTGTTA |
| REN | CTGTCATCTATGCCTTTGTTGGA | AGCTTGCACGATCAGGATTGTCT |
| ADA | GCTGCCACAGGAGTCAAAGT | GCCATGAAGAGGGCAATGTG |
| PNP | TCTACGCAGTGCTTCTTTGC | AGCAGGTGACTGGAGCCTTA |
| PYGM | GTTGGGTTTGGAGTGCTGATT | GGGCATCCCAGCAATAACCA |
| PYGL | CTGCTATCAGTCATCGGCTTTG | AGAGATGAGGATAACAGTGAAGCC |
| PYGB | GCCATTGACGGACAGTGAGA | TCCTTGACCAGCGTGAAGTG |

**Table S4. List of the compounds identified**

| **No.** | **Time（min）** |  | **m/z** | **m/z** | **ppm** | **Formula** | **Weight** | **Name** |
| --- | --- | --- | --- | --- | --- | --- | --- | --- |
| 1 | 1.93 | [M-H]^-^ | 290.087 | 290.0881 | -3.9 | C_11_H_17_NO_8_ | 291.10 | N-Fructosyl pyroglutamate |
| 2 | 2.61 | [M-H]^-^ | 243.0627 | 243.0623 | 1.8 | C_9_H_12_N_2_O_6_ | 244.07 | Uridine |
| 3 | 3.38 | [M-H]^-^ | 344.0405 | 344.0402 | 1 | C_10_H_12_N_5_O_7_P | 345.05 | Cyclic guanosine monophosphate Guanosine |
| 4 | 5.66 | [M-H]^-^ | 282.0834 | 282.0844 | -3.5 | C_10_H_13_N_5_O_5_ | 283.09 | Guanosine |
| 5 | 9.08 | [M-H]^-^ | 203.0828 | 203.0826 | 1.0 | C_11_H_12_N_2_O_2_ | 204.09 | L-tryptophan |
| 6 | 9.17 | [M-H]^-^ | 235.1081 | 235.1088 | -3 | C_12_H_16_N_2_O_3_ | 236.12 | Phenylalanylalanine |
| 7 | 11.89 | [M-H]^-^ | 210.0782 | 210.0772 | 4.8 | C_10_H_13_NO_4_ | 211.08 | 3-Methoxy-L-tyrosine |
| 8 | 12.59 | [M-H]^-^ | 385.1128 | 385.114 | -3.2 | C_17_H_22_O_10_ | 386.12 | Sinapoylglucose |
| 9 | 12.59 | [M+FA-H]^-^ | 431.1932 | 431.1923 | 2.2 | C_19_H_30_O_8_ | 386.19 | Komaroveside A or isomer |
| 10 | 12.97 | [M-H]^-^ | 787.1938 | 787.1938 | -0.1 | C_33_H_40_O_22_ | 788.20 | Quercetin 3-sophoroside 7-glucoside |
| 11 | 13.61 | [M+FA-H]^-^ | 449.2016 | 449.2028 | -2.8 | C_19_H_32_O_9_ | 404.20 | Komaroveside C |
| 12 | 14.55 | [M-H]^-^ | 771.1974 | 771.1989 | -2 | C_33_H_40_O_21_ | 772.21 | Kaempferol 3-sophoroside 7-glucoside |
| 13 | 15.95 | [M-H]^-^ | 625.1403 | 625.141 | -1.2 | C_27_H_30_O_17_ | 626.15 | Quercetin-3-O-sophoroside |
| 14 | 16.56 | [M+FA-H]^-^ | 433.208 | 433.2079 | 0.2 | C_19_H_32_O_8_ | 388.21 | Corchoionoside A |
| 15 | 16.91 | [M-H]^-^ | 223.0615 | 223.0612 | 1.4 | C_11_H_12_O_5_ | 224.07 | Sinapic acid |
| 16 | 18.60 | [M-H]^-^ | 609.1451 | 609.1461 | -1.7 | C_27_H_30_O_16_ | 610.15 | Kaempferol-3-O-sophoroside |
| 17 | 19.81 | [M-H]^-^ | 463.0903 | 463.0882 | 4.5 | C_21_H_20_O_12_ | 464.10 | Hyperoside |
| 18 | 23.67 | [M-H]^-^ | 433.1119 | 433.114 | -4.9 | C_21_H_22_O_10_ | 434.12 | Naringenin-7-O-glucoside |
| 19 | 24.51 | [M-H]^-^ | 447.0917 | 447.0933 | -3.5 | C_21_H_20_O_11_ | 448.10 | Astragalin |
| 20 | 33.20 | [M-H]^-^ | 959.2803 | 959.2827 | -2.5 | C_45_H_52_O_23_ | 960.29 | 1,2,2'-Trisinapoylgentiobioside |
| 21 | 35.14 | [M-H]^-^ | 327.2172 | 327.2177 | -1.5 | C_18_H_32_O_5_ | 328.23 | Trihydroxyoctadecadienoic acid |
| 22 | 35.74 | [M-H]^-^ | 329.2328 | 329.2333 | -1.7 | C_18_H_34_O_5_ | 330.24 | Trihydroxyoctadecenoic acid |

**Table S5. Effects of CCE on levels of** **XOD on hyperuricemic rats (n = 8).**

| Group |  | XOD(U/L) |
| --- | --- | --- |
| Control |  | 16.88±1.54 |
| Model | 1000mg/kg | 18.90±1.93^#^ |
| PG | 27mg/kg | 16.69±2.23* |
| L-CVE | 0.75g/kg | 18.35±1.33 |
| M-CVE | 1.5g/kg | 18.05±1.66 |
| H-CVE | 3g/kg | 19.15±1.51 |

Note: Compared with the Control group: ^#^*p* < 0.05; Compared with the Model group: **p* < 0.05.

Data are expressed as means ± SEM.

**Figure S1. Gene–metabolite networks by MetaboAnalyst.**


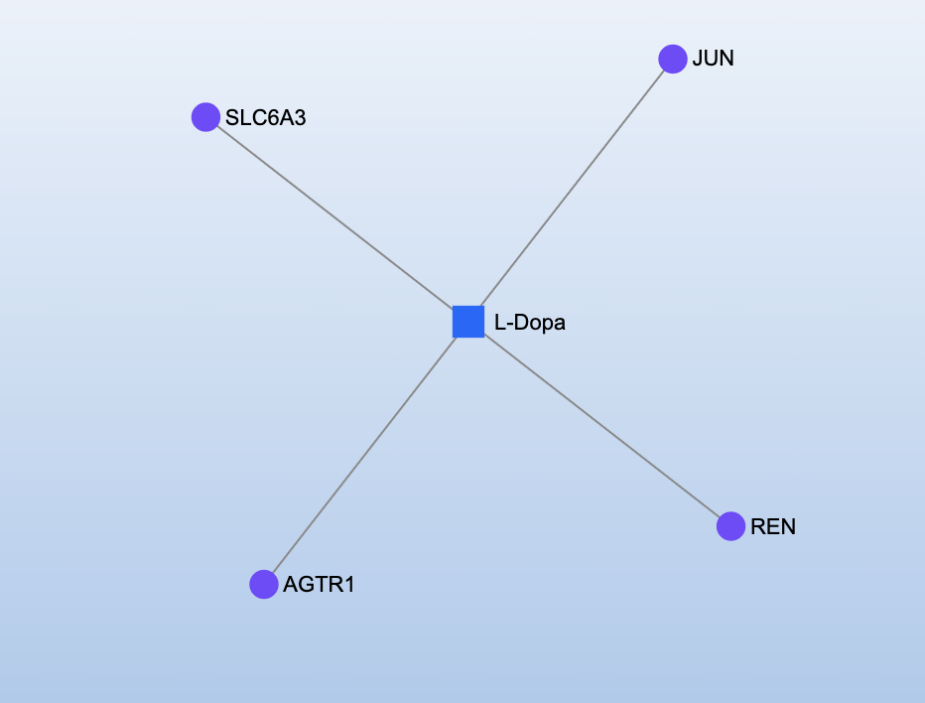


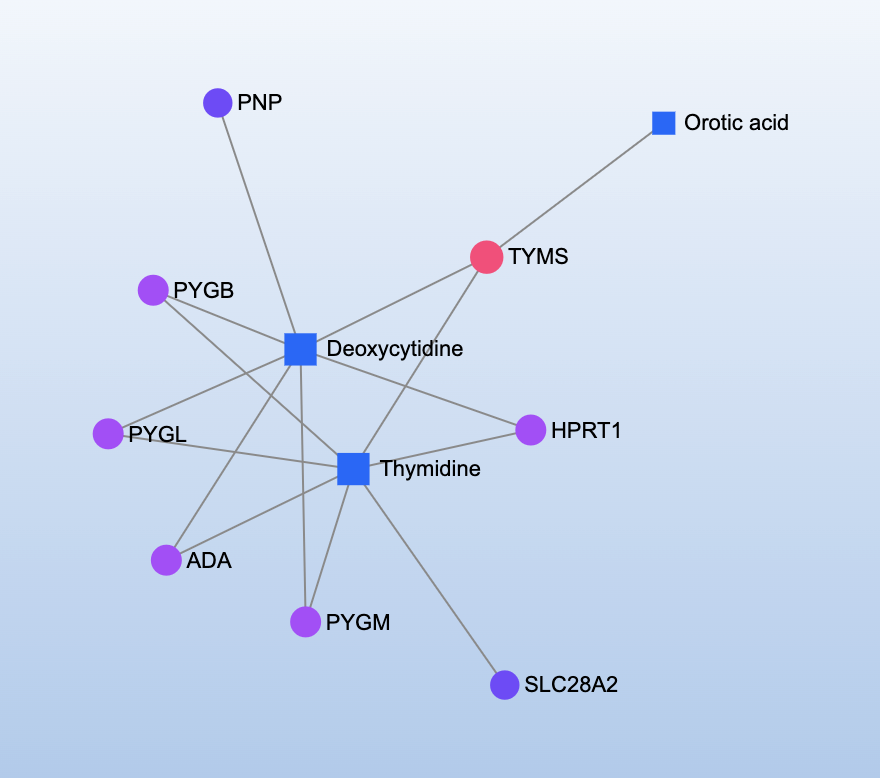

Supplement: Supplementary file 2 [file DataSheet2.DOCX]
